# Stroke in Brazil: prevalence, activity limitations, access to healthcare, and physiotherapeutic treatment

**DOI:** 10.1055/s-0044-1792094

**Published:** 2024-12-20

**Authors:** Luana Karoline Castro Silva, Cristian Douglas Dantas de Sousa, Ramon Távora Viana, Renata Viana Brígido de Moura Jucá, Johnnatas Mikael Lopes, Christina Danielli Coelho de Morais Faria, Shamyr Sulyvan de Castro, Lidiane Andrea Oliveira Lima

**Affiliations:** 1 Universidade Federal do Ceará, Programa de Pós-Graduação em Fisioterapia e Funcionalidade, Fortaleza CE, Brazil.; 2 Universidade Federal do Ceará, Departamento de Fisioterapia, Fortaleza CE, Brazil.; 3 Universidade Federal do Vale do São Francisco, Curso de Medicina, Paulo Afonso BA, Brazil.; 4 Universidade Federal de Minas Gerais, Programa de Pós-Graduação em Ciências da Reabilitação, Belo Horizonte MG, Brazil.

**Keywords:** Stroke, Activities of Daily Living, Health Services Accessibility, Population Studies in Public Health, Health Surveys, Acidente Vascular Cerebral, Atividades Cotidianas, Acessibilidade aos Serviços de Saúde, Estudos Populacionais em Saúde Pública, Inquéritos Epidemiológicos

## Abstract

**Background** Stroke remains a public health problem, reported as the third cause of disability. Among survivors, the ability to perform usual daily activities may be reduced, requiring rehabilitation.

**Objective** To investigate the prevalence of self-reported stroke, the accessibility of healthcare, and the degree and percentage of patients with limitations in usual activities who are unassisted by physiotherapeutic treatment in different regions of the country.

**Methods** This cross-sectional study was conducted using data from the 2019 National Health Survey. Participants aged 15 years or older from all five geographic regions of Brazil who reported a diagnosis of stroke were included. The data were analyzed using sample weighting and expressed as estimates along with a 95% confidence interval (CI).

**Results** The national prevalence of self-reported stroke in Brazil was 1.9% (95%CI 1.7–2.0), equivalent to 1,975 individuals with diagnosis. Of these, 50.2% reported limitations in their daily activities, and more than half (54.6%) had regular follow-ups with healthcare professionals. However, only 24.6% reported having access to rehabilitation, while 73.4% of individuals with activity limitations received no physiotherapeutic treatment.

**Conclusion** The prevalence of' self-reported stroke in the Brazilian population was 1.9%, with more than half experiencing limitations in their activities. While more than half of the stroke patients underwent follow-ups from a health professional, only ¼ of them reported having access to rehabilitation. Government interventions are necessary to ensure effective access to healthcare, including rehabilitation for the Brazilian population.

## Resumo

**Antecedentes** O acidente vascular cerebral (AVC) continua sendo um problema de saúde pública, relatado como a terceira causa de incapacidade. Entre os sobreviventes, a capacidade de realizar atividades diárias habituais pode ser reduzida, demandando reabilitação.

**Objetivo** Investigar a prevalência de AVC autorreferido, a acessibilidade aos cuidados de saúde, assim como o grau e porcentagem de pacientes com limitações nas atividades habituais que não são assistidos por tratamento fisioterapêutico em diferentes regiões do país.

**Métodos** Estudo transversal realizado com dados da Pesquisa Nacional de Saúde de 2019. Foram incluídos participantes com 15 anos ou mais de todas as cinco regiões geográficas do Brasil. Os dados foram analisados por meio de ponderação amostral e expressos como estimativas com intervalo de confiança (IC) de 95%.

**Resultados** A prevalência nacional de AVC autorreferido no Brasil foi de 1,9% (IC95% 1,7–2,0), equivalente a 1.975 indivíduos com diagnóstico. Destes, 50,2% relataram limitações em atividades diárias e mais da metade (54,6%) faziam acompanhamento regular com profissionais de saúde. Contudo, apenas 24,6% relataram acesso à reabilitação, enquanto 73,4% dos indivíduos com limitação de atividades não receberam tratamento fisioterapêutico.

**Conclusão** A prevalência de AVC autorreferido no Brasil foi de 1,9%, com mais da metade apresentando limitações em atividades. Embora mais de metade dos pacientes recebessem acompanhamento de um profissional de saúde, apenas ¼ deles relataram ter acesso à reabilitação. Intervenções governamentais são necessárias para garantir o acesso efetivo à saúde, incluindo a reabilitação, para a população brasileira.

## INTRODUCTION

Stroke continues to be a significant public health concern worldwide. According to the Global Burden of Disease in 2019, it is the second leading cause of death and the third leading cause of disability, as measured by disability-adjusted life years (DALYs). 1 In recent decades, high-income countries have managed to decrease the incidence of strokes due to health policies that aim to reduce risk factors. 2 In low- and middle-income countries, there is a growing proportion of the incidence and an increasing burden of morbidity. 3 In Brazil, stroke has once again become the leading cause of mortality after 7 years in second place, according to data from the Transparency Portal of the Civil Registry Offices. 4 According to the Brazilian National Health Survey (PNS), in 2013, there was a 1.5% incidence of stroke or cerebrovascular accident diagnosis, representing approximately 2.2 million people aged 18 or over. 5

In 2024, DATASUS-tabnet reported varying numbers of hospitalizations due to stroke across different regions. It's important to note that the information may be biased due to underreporting. However, it provides valuable data for objective analysis of the Brazilian population's health status. This data allows for the monitoring of health indicators on an annual basis. For instance, in 2023, there was a mortality rate of 15.9% due to stroke in the northeast of the country, compared to 11.5% in the south region. In the Matão stroke registry study, in the state of São Paulo, in the southeast region, from 2003 to 2004 and 2015 to 2016, the stroke incidence decreased by 39% and mortality by 50%. 6

The survival rate for stroke patients varies across different segments of the population, depending on the level of technological advancements and the consequent improvements in healthcare. If current trends persist, by 2050, there will be more than 200 million stroke survivors. 7 8 It is estimated that ⅓ of survivors will experience permanent disabilities for the rest of their lives. Additionally, approximately 50% of them require assistance with daily activities, while 70% are unable to resume their previous work activities. 9

The growing number of stroke survivors facing functioning loss, along with disability, limited activity, or reduced participation highlights the rising necessity of medical intervention poststroke. 10 11 Immediate access to rehabilitation professionals during and after hospitalization is recommended for individuals to take advantage of the peak of neuroplasticity in the first months following the event. 12 13

The Stroke Care Line in Brazil recommends that every patient be referred to a multidisciplinary team for poststroke planning, undergo functional reassessment in primary healthcare, and have regular follow-ups every 6 months at most. However, rehabilitation, defined as a set of interventions aimed at optimizing functioning and promoting participation in individuals' daily living activities, 14 varies in its implementation by region.

In most parts of the country, stroke survivors have limited opportunities to receive therapy after the acute phase. In public healthcare centers, patients are discharged and referred to primary services, with the availability of rehabilitation depending on the public healthcare system. On the other hand, through private healthcare insurance, stroke patients are sent to rehabilitation centers upon discharge and receive immediate support. 15 16 According to Martins et al., 16 after discharge, 54% of stroke patients with some degree of limitation had access to physiotherapy treatment in the public health care, compared with 71.4% in the private sector.

The rehabilitation initiative of 2030 by the World Health Organization (WHO) aims to make this an essential health service and a key component of Universal Health Coverage, represented by the Brazilian Unified Health System (SUS). 14 The SUS is organized in a regionally and hierarchically, with three levels of healthcare complexity. Primary Care is the initial part of ongoing healthcare, complemented by specialized actions. However, the overemphasis on specialized care is still a problem in Brazil, particularly in the private sector.

The National Health Policy for People with Disabilities aims to ensure the rights of this population, emphasizing universal access to healthcare, health equity, and accessibility. This policy ensures direct access to healthcare based on each person's specific needs, guaranteeing accessibility. 17 It is necessary to understand that Brazil has both public and private health systems. However, there are still challenges with integrating these systems and a tendency for healthcare to be more specialized, which can affect the overall quality and effectiveness of the network. This can lead to increased costs and unequal access to health services. 18 Private health insurance coverage is lower in the North (the country's poorest region) and highest in the South (the richest). 19 Approximately 70% of the Brazilian population relies on the public health system. 20 A study by Magalhães et al. 21 found that most initial posthospital discharge stroke consultations were provided by public services.

Considering how the high morbidity burden and disability caused by stroke and the limited access to rehabilitative assistance can exacerbate its effects, national population-based analyses of healthcare for stroke patients can guide the development and enhancement of poststroke healthcare policies. This study aimed to:

- Investigate the prevalence of self-reported stroke and degrees of limitations in usual activities in Brazil;
- Estimate the prevalence of healthcare accessibility for poststroke patients in the country's different geographic regions;
- Verify the percentage of patients with limitations in their usual poststroke activities who remained unassisted by physiotherapeutic treatment in different regions of the country.

## METHODS

This study adhered to the cross-sectional reporting guidelines established by STROBE (Strengthening the Reporting of Observational Studies in Epidemiology).

### Design

This study is a cross-sectional, population-based survey using data from the 2019 PNS, which is a national health survey conducted by the Ministry of Health, in collaboration with the Brazilian Institute of Geography and Statistics (IBGE) to gather data on the performance of the national health system, including the access to and utilization of health care, continuity of care, the health status of the population, and the surveillance of chronic noncommunicable diseases and associated risk factors. The PNS data are available online on the IBGE website. 22

### Population, sample, and sampling

The PNS participants were selected through complex sampling by conglomerates in the country's five regions. The process was carried out in three stages: first, the selection of census sectors; second, the selection of households; and, finally, the random selection of a resident over 15-years-old from each household. Some excluded households were located in areas with sparse populations, Indigenous groups, military bases, camps, boats, prisons, elderly care facilities, hospitals etc.. Further details can be found in publications by Stopa et al. and IBGE. 23

The population for this study included all individuals over the age of 15 who participated in the PNS survey, which had a response rate of 90,846. The sample for this study consisted of all participants, of both sexes, residing in permanent private homes located in urban and rural areas of all Federation Units. They were required to report having received a medical diagnosis of stroke, which was determined by asking: “Has any doctor ever diagnosed you with a cerebrovascular accident or stroke?”

### Collection procedure

The PNS 2019 data collection was conducted by IBGE through a structured questionnaire with identification questions, followed by 22 modules (named by letters of the alphabet, module A–V) consisting of 80 pages. Trained interviewers used computer-assisted personal interviews and visited households. The health interview manual provided to interviewers contains information about the objectives of each question, recommendations, potential answers, and observations. The interviews were conducted with a household resident providing information. 24

### Collected variables

The study focused on several areas, including sociodemographic characteristics, stroke prevalence, self-reported limitations in usual activities, and access to healthcare for individuals diagnosed with a stroke.

- Sociodemographic characteristics: sex (female or male); age (years); age at first stroke, divided by age group (<18, 18–50, >50 years); stroke time (years); spouse or partner (yes or no); color or race (White, Black, Yellow, Brown, or Indigenous) and educational level (illiterate, elementary I, elementary II, high school, higher education). 25
- Prevalence of stroke: Numerator Q068: “Has a doctor ever diagnosed you with a cerebrovascular accident or stroke?” Denominator: 1. Yes or 2. No. 25 (Note: self-reported measures introduce subjectivity in responses and memory bias commonly associated with this type of measurement).
- Activity performance through self-reported limitations in usual activities. Numerator Q73: “In general, to what degree does the stroke limit your usual activities (such as working, doing household chores etc.)?” Denominators: 1. Does not limit; 2 Limits a little; 3. Limits moderately; 4. Limits intensely; and 5. Limits very intensely. 25 (Note: the questionnaire does not allow identifying regularity in the degree of limitation due to a stroke).
- Access to healthcare:
  ▪ Numerator Q07213: “Due to the stroke, are you regularly monitored by a health professional?” Denominator: 1. Yes or 2. No; (Note: the questionnaire does not allow for detailed identification of the specific health professional the individual refers to for regular follow-up).
  ▪ Numerator Q07209: “Do you currently receive physiotherapy due to the stroke?” Denominator: 1. Yes or 2. No (Note: the questionnaire does not allow identifying the period of initiation of physiotherapeutic treatment after the stroke event).
  ▪ Numerator Q07210: “Do you currently receive other rehabilitation therapies due to the stroke?” Denominator: 1. Yes or 2. No (Note: the questionnaire does not allow identifying in detail which other rehabilitation professionals the individual refers to).

### Data analysis

The data analysis took into account the sampling weight of the participants, as complex sampling was used to select them. The absolute and relative frequencies of the variables were calculated to determine the range and interval measurements. These were displayed as a 95% confidence interval (95%CI) to illustrate the variability of the events. All measurements were analyzed by geographic region of Brazil, and statistical analysis was conducted using the Statistical Package Social Sciences (SPSS, IBM Corp., Armonk, NY, USA) software, version 22, for complex sample modules.

## RESULTS

**Figure 1:**
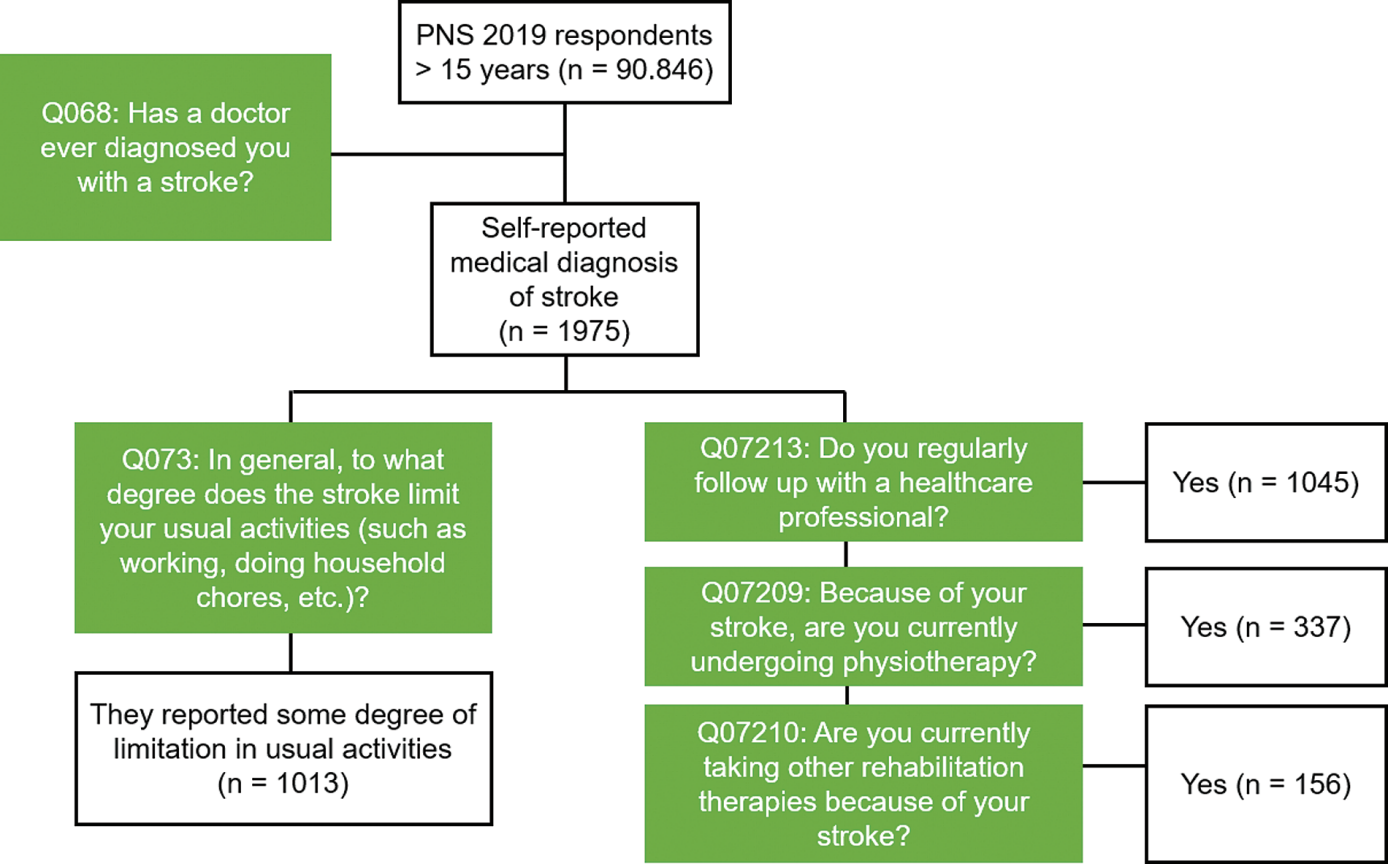
Research flowchart with the initial number of participants, response rates, and flow of questions regarding the self-reported stroke diagnosis and follow-up in healthcare.

Our results highlight the prevalence of self-reported stroke diagnosis and access to poststroke healthcare. Figure 1 displays the research flowchart showing the number of participants, response rates, and question flow.

**Figure 2:**
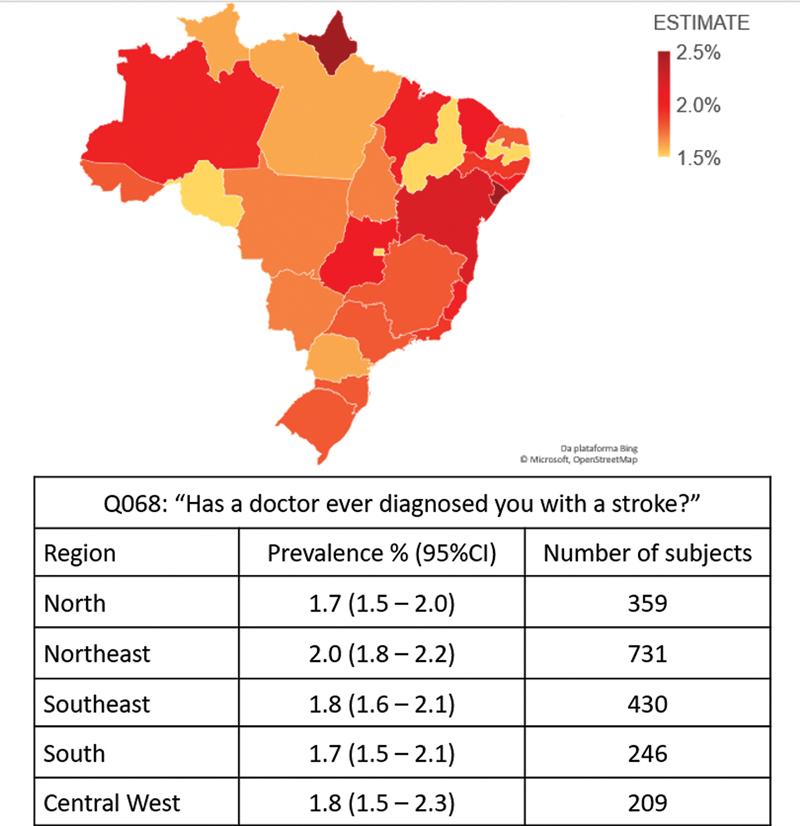
Geographic representation of the prevalence of stroke cases in Brazil.

In 2019, 1.9% (95%CI: 1.7–2.0) of individuals aged 18 years or older were diagnosed with a stroke out of 90,849 people analyzed in the PNS. When observed by region, the highest prevalence was in the Northeast (2.0%; 95%CI: 1.8–2.2) and the lowest in the South (1.7%; 95%CI: 1.5–2.1), without statistical significance between regions. Figure 2 shows the regional prevalence of stroke in Brazil and presents a graphical representation of self-reported stroke diagnoses in all of Brazil.

The clinical and sociodemographic information of poststroke individuals is described in Table 1 . The reported performance data indicates that 50.2% of individuals who have had a stroke experienced some level of limitation in their daily activities, ranging from slight to very intense. These limitations varied across the regions, with a statistically significant difference observed between the Northeast and Southeast. The Southeast had the highest rate of individuals without activity limitation (59.2%, 95%CI: 51.1–66.7), while the Northeast had the lowest (43.9%; 95%CI: 38.9–49.0). Although there was no statistical difference in the degrees of limitation between them, there were significant differences in the degrees of limitation within the same region. Both the Northeast and South had more individuals with intense or very intense (Northeast: 23.6%, 95%CI: 19.4–28.4; South: 21.6%, 95%CI: 16.8–27.4) than moderate (Northeast: 12.9%, 95%CI: 9.8–16.4; South: 11.3%, 95%CI: 7.8–16.0) limitations. Meanwhile, the North had more individuals with little (25.4%, 95%CI: 19.2–32.8) than moderate (11.1%, 95%CI 7.5–16.1) limitation. For further details on the degrees of limitation in poststroke activities across regions of Brazil, refer to Table 2 .

**Table 1:** Sociodemographic characteristics of individuals after a stroke by geographic region of Brazil

Variable		Region, % (95%CI)				
		North	Northeast	Southeast	South	Midwest
**Sex**	Female	55.5 (48.2–62.5)	53.2 (47.8–58.4)	52.6 (45.2–59.8)	53.7 (46.0–61.2)	54.0 (43.6–64.2)
	Male	44.5 (37.5–51.8)	46.8 (41.6–52.2)	47.4 (40.2–54.8)	46.3 (38.8–54.0)	46.0 (35.8–56.4)
**Age (years)**		61.1 ± 1.2	63.2 ± 0.8	64.5 ± 1.6	63.5 ± 1.0	57.3 ± 1.6
** Age of 1 ^st^ stroke **	< 18	8.9 (5.4–14.2)	3.5 (2.3–5.4)	3.1 (1.8–5.5)	2.1 (0.9–4.8)	4.8 (1.9–11.2)
	18–50	41.8 (34.6–49.4)	36.6 (32.3-41.2)	35.2 (28.1–43.1)	31.5 (25.3–38.4)	46.5 (37.9–55.4)
	> 50	49.3 (42.4–56.3)	59.8 (55.2–64.3)	61.6 (53.9–68.8)	66.4 (59.5–72.8)	48.7 (40.0–57.5)
**Stroke time (years)**		11.6 ± 0.8	9.9 ± 0.5	9.7 ± 0.7	8.7 ± 0.7	8.0 ± 0.8
**Spouse or partner**	Yes	62.2 (55.9–68.2)	58.0 (52.3–63.5)	50.8 (43.7–57.8)	65.8 (57.4–73.3)	60.4 (50.7–69.3)
	No	37.8 (31.8–44.1)	42.0 (36.5–47.7)	49.2 (42.2–56.3)	34.2 (26.7–42.6)	39.6 (30.7–49.3)
**Color or race**	White	20.3 (15.0–26.9)	27.7 (22.5–33.6)	43.5 (37.2–49.9)	76.7 (69.7–82.4)	32.9 (24.7–42.3)
	Black	13.4 (8.3–20.9)	14.0 (11.2–17.4)	14.7 (10.9–19.6)	8.2 (4.0–15.9)	8.2 (4.8–13.8)
	Yellow	0.3 (0.1–1.7)	0.6 (0.2–2.5)	1.7 (0.9–3.2)	0.7 (0.2–3.3)	2.3 (0.8–6.2)
	Brown	65.0 (57.5–71.8)	56.8 (51.1–62.2)	39.5 (32.9–46.6)	14.3 (10.2–19.6)	56.1 (46.4–65.4)
	Indigenous	0.9 (0.4–2.2)	0.9 (0.5–1.5)	0.5 (0.2–1.8)	0.2 (0.0–1.3)	0.5 (0.1–3.3)
**Schooling** **(years)**	≤ 4	33.7 (26.6–41.6)	43.5 (37.9–49.2)	39.8 (33.5–46.5)	41.3 (33.9–49.1)	37.9 (27.9–49.1)
	5–8	31.9 (24.6–40.1)	30.0 (24.9–35.8)	30.7 (25.1–37.1)	30.5 (22.7–39.7)	28.0 (21.0–36.3)
	9–11	21.0 (15.2–28.3)	18.4 (14.8–22.6)	20.6 (16.1–26.0)	16.8 (12.0–22.9)	23.3 (15.2–34.0)
	≥ 12	13.4 (8.6–20.3)	8.1 (6.3–10.3)	8.9 (6.1–12.7)	11.4 (7.5–17.0)	10.8 (7.0–16.3)

Abbreviation: 95%CI, confidence interval.

**Table 2:** Prevalence of the degree of self-reported limitation in usual activities after stroke in the Brazilian population

Degree of activity limitations resulting from the stroke	Region, % (95%CI)				
	North	Northeast	South	Southeast	Midwest
None	46.2 (39.4–53.1)	43.9 (38.9–49.0)	51.5 (44.8–58.1)	59.2 (51.1–66.7)	49.0 (39.5–58.5)
Little	25.4 (19.2–32.8)	19.7 (15.7–24.5)	15.6 (10.4–22.7)	13.9 (9.3–20.4)	21.2 (13.8–31.2)
Moderate	11.1 (7.5–16.1)	12.8 (9.8–16.4)	11.3 (7.8–16.0)	8.6 (5.0–14.3)	13.9 (10.1–18.8)
Intense or very intense	17.3 (12.8–23.2)	23.6 (19.4–28.4)	21.6 (16.8–27.4)	18.3 (13.3–24.7)	15.9 (11.5–21.6)

Abbreviation: 95%CI, confidence interval.

**Figure 3:**
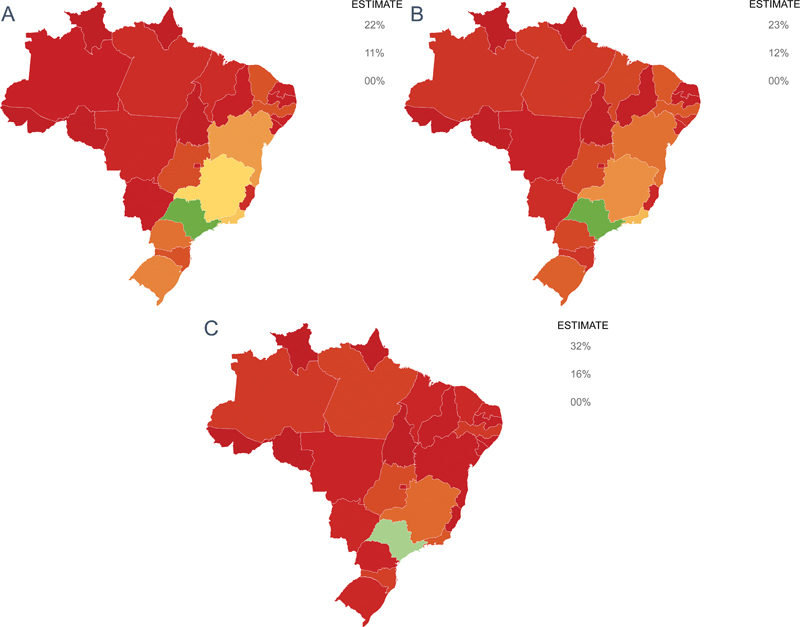
Geographic representation of access to post-stroke healthcare in Brazil. ( **A** ) Regular follow-up with a healthcare professional. ( **B** ) Use of physiotherapy services. ( **C** ) Use of other rehabilitation services.

In Brazil, only 54.6% of poststroke patients receive regular monitoring from a health professional. When specifically asked about follow-up with a rehabilitation professional, 16.7% underwent physiotherapeutic treatment, and merely 7.9% received other rehabilitation therapies. This resulted in only approximately ¼ (24.6%) of poststroke individuals engaged in rehabilitation activities. The study also offers a comprehensive overview of healthcare accessibility following a stroke in different geographical regions of Brazil ( Table 3 ). The frequency of follow-up visits with healthcare professionals varied across regions. The North and Northeast had lower follow-up rates (North: 39.4%, 95%CI: 32.7–46.5; Northeast: 48.7%, 95%CI: 43.5–53-9) compared to the South (65.3%, 95%CI: 56.8–72.9). Additionally, the North had lower follow-up rates than the Southeast (56.8%, 95%CI: 48.8–64.5) and Central-West (59.5%, 95%CI: 51.4–67.1). No significant differences were found among regions in terms of restricted access to physiotherapy and other rehabilitation therapies. Furthermore, Figure 3 provides a visual representation of healthcare accessibility across all federative units in Brazil.

**Table 3:** Percentage of access to poststroke healthcare in geographic regions in Brazil, with respective visualization of the estimated 95%CI for the adult population

Access to healthcare poststroke (answer = yes)	Region, % (95%CI)				
	North	Northeast	Southeast	South	Midwest
Regular follow-up with a healthcare professional	39.4 (32.7–46.5)	48.7 (43.5–53.9)	56.8 (48.8–64.5)	65.3 (56.8–72.9)	59.5 (51.4–67.1)
Physical therapy	17.4 (12.5–23.8)	16.5 (13.1–20.6)	16.7 (11.3–24.0)	14.4 (10.1–20.3)	21.0 (13.9–30.5)
Other rehabilitation therapies	10.9 (6.7–17.2)	5.5 (4.0–7.6)	8.8 (4.3–16.9)	5.7 (3.8–8.5)	12.8 (7.1–22.0)

Abbreviation: 95%CI, confidence interval.

Only 26.6% (95%CI: 21.7–32.1) of stroke patients who reported having some degree of activity limitation received physiotherapy. The regional analysis of this data shows that there are no significant differences in access to physiotherapy for people with activity limitations in Brazil. In all regions, there is limited access to physiotherapy for individuals with functional limitations, with more than 70% of them not receiving this treatment.

These findings underscore the need to monitor regional disparities in access to healthcare and to promote equity in the health system, particularly for the most vulnerable populations. The data suggests that patients with activity limitations after a stroke face challenges in accessing physiotherapeutic treatment across Brazil. Detailed information on the percentage of patients in each region is provided in Table 4 .

**Table 4:** Percentage of patients with limitations in usual activities who are unassisted by poststroke physiotherapeutic treatment in the geographic regions of the country

**Does not do physical therapy** **% (95%CI)**	**Region**	**Limitation of activities resulting from stroke** **% (95%CI)**	
		**No**	**Yes**
	**North**	93.0 (88.2–96.0)	73.6 (63.2–82.0)
	**Northeast**	92.9 (89.5–95.2)	76.1 (69.4–81.8)
	**Southeast**	94.1 (90.0–96.6)	71.8 (59.9–81.3)
	**South**	94.4 (87.4–97.6)	72.7 (61.7–81.5)
	**Midwest**	86.7 (66.4–95.5)	71.6 (61.2–80.1)

Abbreviation: 95%CI, confidence interval.

## DISCUSSION

This is the first population-based study that explored the prevalence of self-reported stroke, the degree of limitation in usual activities, access to healthcare, and lack of physiotherapeutic treatment among individuals who had a stroke in all five regions of the country. According to the 2019 PNS, which surveyed a representative sample of the Brazilian population, the prevalence rate of stroke was 1.9%. This study also revealed that most individuals who suffered from a stroke experienced some degree of activity limitation and continued to receive regular medical attention. However, only a small portion of these individuals who reported limitations in activity received physiotherapeutic treatment, regardless of the region. Around 73.4% were not undergoing rehabilitation therapies poststroke.

Across the country, stroke prevalence estimates differ slightly between studies because each study selects and recruits a sample of participants to represent the target study population (e.g., state, region, or country). 26 In community-based studies in the city of São Paulo (Southeast), from 2011, the age-adjusted prevalence rate for men was 4.6% and for women 6.5%, 27 while, in another study in the same city, a prevalence of 6.8% of stroke survivors was identified. 28 In the town of Coari (North), authors have found a crude prevalence of stroke of 6.3% in rural areas and of 3.7% in urban areas. 29 These studies are not able to measure the magnitude of stroke prevalence on a national level, so information from the nationwide surveillance system must be reported. Therefore, the present study, unlike other studies with representative data from some regions of the country, provides data on the epidemiology of stroke with a national scope. In this study, it was found that the national prevalence of stroke was 1.9%, higher than the rate reported in PNS 2013 (1.5%).

From a nationally representative population-based epidemiological survey, the prevalence of stroke in Brazil is similar to rates in other low- to middle-income countries. In contrast, high-income countries, such as Canada, reported a prevalence rate of 1.1% in 2013, 30 and estimated an increase in the prevalence rate of 1.5 to 1.6% by 2038. 30 Health inequalities are often associated with income disparities and can significantly affect a population's health. For instance, the stroke prevalence in Brazil in 2019 is higher than the estimated prevalence in Canada in 2038. In Australia, the prevalence decreased from 1.7 in 2013 to 1.3% in 2018, 31 highlighting the connection between the country's economy and stroke rates. Therefore, the high prevalence of stroke in Brazil highlights the need for effective measures to reduce its impact on the healthcare system and the population. To achieve this, the government should promote prevention activities, such as encouraging a healthy lifestyle, along with discouraging sedentary habits and promoting a balanced diet. 32 Early detection of genetic factors, as well as control of diseases such as hypertension, diabetes, and obesity, should also be prioritized. These measures can help minimize the prevalence of stroke and its adverse effects on the population.

Poststroke disabilities, characterized by activity limitations, were observed in the majority of individuals in our sample (50.2%). This observation is supported by the fact that stroke is still the second leading cause of DALYs' data among chronic noncommunicable diseases. 33 Additionally, stroke is the main cause of years lived with disability (YLDs) in the country. 34

The prevalence of limitations is observed in both low- and high-income countries. In Australia, 64% of individuals living with stroke reported some form of limitation in 2022 according to the Australian Institute of Health and Welfare. 35 This means that a stroke can cause disability and limitations, irrespective of the income level of the country. In Brazil, there is a noticeable difference in the prevalence of limitations within different regions, particularly between areas with varying economic status. For example, the Southeast region has a higher rate of individuals without activity limitations compared to the Northeast region, which is considered the poorest region in the country. Therefore, when it comes to stroke rehabilitation, it's crucial to ensure that it aligns with the principle of fairness upheld by the Brazilian unified health system (SUS). These services should not only be readily available but also consider in which regions of the country individuals face more limitations. This approach should also consider the varying degrees of limitations as, even within the same region, there may be individuals with more severe limitations, particularly in the Northeast and Southeast regions.

Our results demonstrated that regular follow-up with a health professional showed differences between regions, where individuals from poorer regions of the country had shorter follow-up times than individuals from regions with higher income. However, even regions with the highest follow-up times maintained restricted access to physiotherapy and other rehabilitations. This suggests that poststroke follow-up by a healthcare professional did not result in access to physiotherapeutic treatment and other rehabilitation therapies. Only about a quarter of the individuals in the study received rehabilitation services.

The World Stroke Organization's (WSO) guideline defines ideal care of stroke patients across the continuum and addresses that models for stroke services delivery vary considerably from region to region, and greatly depend on the availability of resources. Resource availability impacts the extent to which comprehensive stroke care can be provided across the continuum, from acute stroke management to rehabilitation, prevention of recurrence, community reintegration, and long-term recovery. 36 In agreement with our findings, Silva et al., 37 in a study on stroke care services in Brazil, states that it has undergone a major change in the last decade to adequately plan a global change, starting with primary prevention and including acute stroke care and rehabilitation services. However, Brazil is still a country with great social inequalities and there is still a lot to be implemented.

This study found that access to rehabilitation services in Brazil is limited for most of the population, regardless of the region. The rate is similar to that of low- and middle-income countries, but different from high-income ones. For example, in Nigeria and South Africa, the access rates are only 25.2 and 14%, respectively, while in Canada and Australia, they were 81 and 61%, respectively. This data suggests that public and private healthcare systems are influenced not only by biological determinants but also by social and economic factors that impact people's health, both individually and collectively. Therefore, improving the socioeconomic conditions of the country is crucial for increasing access to rehabilitation for stroke patients.

Finally, individuals with activity limitations in Brazil lack proper physiotherapeutic treatment nationwide, irrespective of macro-regional differences. This information highlights the inequality and inequity in accessing post-stroke rehabilitation services. It is important to note that such a lack of assistance should not occur in terms of public health policies, since the SUS is guided by principles of comprehensiveness and equity in health, among others. 38 This means that every person has the right to receive healthcare meeting their individual needs. The National Health Policy for People with Disabilities, last updated in 2023, reinforces SUS's principles; 15 and the established care line provides rehabilitation services for patients with chronic stroke in the primary health care unit (UPAS). 39 Despite the existence of policies and strategies for stroke patients' healthcare in Brazil, it still hasn't met those recommended standards.

This study raises important questions regarding poststroke rehabilitation in Brazil. The results indicate that our country faces more challenges compared to high-income ones, yet we address them similarly to low- and middle-income countries. Therefore, it is urgent to identify and understand the specific barriers that prevent effective multidisciplinary in-hospital care in Brazil. 40 Strategies to mitigate the loss of the therapeutic window of neuroplasticity should be a priority for public policies, as it optimizes functional recovery and helps prevent disabilities and severe activity limitations poststroke. Finally, to meet the demands of WHO's proposal for unrestricted access to rehabilitation by 2030, the question of why it is rarely prioritized should be addressed.

Despite the important results, this study had some limitations. Self-reported measures present subjectiveness to responses, in addition to the associated risk of memory bias. Still, reporting these findings is relevant, as they reflect the perception of a substantial and representative sample of the Brazilian population.

## CONCLUSION

The high prevalence rate of stroke among the majority of individuals who developed disabilities, exacerbated by limited access to rehabilitation therapies, composes the portrait of stroke and the available assistance in Brazil. Although the majority of individuals have sought health professional attention after experiencing a stroke, those who have reported limitations in activity are the most affected in terms of accessing physiotherapeutic treatment. Healthcare facilities that specialize in treating strokes need to make sure that rehabilitation services are readily available or easily accessible. It's also important to raise awareness about comprehensive stroke centers and improve their accessibility. Researching the factors that limit activity and access to rehabilitation, as well as conducting cost-effectiveness studies of post-stroke rehabilitation, can support the development of public policies and effective actions to improve stroke care and address existing inequalities.
